# Heterogenous COVID-19 transmission dynamics within Singapore: a clearer picture of future national responses

**DOI:** 10.1186/s12916-020-01625-7

**Published:** 2020-06-02

**Authors:** N. Bagdasarian, D. Fisher

**Affiliations:** 1 Department of Medicine, University Medicine Cluster, National University Hospital, Singapore, Singapore; 2 Department of Medicine, Yong Loo Lin School of Medicine, National University of Singapore, Singapore, Singapore

**Keywords:** COVID-19, Transmission dynamics

## Background

In their paper entitled “Real-time monitoring the transmission potential of coronavirus disease 2019 (COVID-19) in Singapore, March 2020”, Tariq and colleagues utilise publicly available data published by the Singapore Ministry of Health to estimate the effective reproduction number (Rt) of COVID-19 in Singapore [1]. The authors evaluate each linked cluster of cases, study transmission chains and take into account estimates of reporting delays. They use this information to calculate an Rt of less than 1.0 and conclude that estimates for Singapore are substantially lower than those reported in other parts of the world due to robust containment efforts in Singapore. The authors point to super-spreader events and asymptomatic shedding as major drivers of transmission heterogeneity in countries like Singapore and South Korea and conclude that maintaining social distancing and active case finding are key to “stomp [sic] out all active or incoming chains of transmission”. However, it is important to centre this analysis in the current situation in Singapore. We must stress that, while the authors’ conclusions are accurate, they do not account for the recent transmission dynamics experienced in Singapore.

## The current situation in Singapore

As of this writing (May 8, 2020), Singapore has had over 20,000 cases of COVID-19, with recent growth rates reaching over 1300 new cases per day [2]. This growth has been predominantly driven by cases coming from migrant workers, living in large, population-dense facilities [2]. More than 20% of Singapore’s population of 5.7 million are foreign workers, and the majority are manual workers from Bangladesh and India, working in construction, shipping, manufacturing and domestic service sectors [3]. Many live in massive dormitory facilities, with the largest having a capacity of 25,000 residents in a single compound, and up to 20 residents sharing a room, with communal bathroom, dining and recreation facilities [3]. This type of crowding provides a very vulnerable setting for infectious disease outbreaks, and the extent of this is now being realised in Singapore, with almost all the recent reported cases of COVID-19 (> 95%) reported from the dormitories [2].

## Multiple outbreaks taking place simultaneously

While the results of the analysis by Tariq et al. are important, the recent developments in Singapore highlight the fact that heterogeneity in an outbreak is not just a possibility, but an inevitability. It appears at first glance that Singapore is experiencing three outbreaks, which can be confirmed by Singapore Ministry of Health data [2] and demonstrated by multiple distinct epidemic curves. The first was an epidemic of imported cases that peaked on March 23rd with less than 50 cases per day; this epidemic curve was abruptly halted by travel restrictions for short-term visitors [4]. Next came an epidemic curve of community cases that peaked on April 8th with less than 60 cases reported per day; this curve saw a sharp decline after a nationwide “circuit breaker” was announced, which closed all non-essential businesses and included social distancing mandates and restrictions on social gatherings, as well as strict penalties for those not complying [5].

However, travel restrictions and social distancing mandates did nothing to prevent a new epidemic of COVID-19 cases from the dormitories, which ballooned just as the strict “circuit breaker” measures were introduced on April 7th, and which peaked on April 20th with 1371 cases from the dormitories reported in a single day [2]. Even this idea of three separate epidemics in Singapore does not tell the whole story. The dormitories themselves are heterogeneous, with each dormitory, each building and each floor undergoing its own small epidemic and revealing its own specific transmission dynamics. Countries will increasingly see that epidemic curves, reproduction numbers and outbreak interventions will vary between contexts within a country.

In the COVID-19 pandemic, heterogeneity in transmission patterns are not the exception, they are the rule, and we will soon see this increasingly identified across the world. Communities may manage to reduce transmission by asking residents to stay at home and comply with social distancing measures, but broadly implemented interventions will not be effective or practical in many settings. COVID-19 outbreaks in the USA in prisons [6] and nursing homes [7] and some highly publicised outbreaks on international cruise ships [8] have already shown us that asymptomatic viral shedding may be the major driver of transmission in crowded conditions where social distancing is not a practical intervention. Outbreaks in three clusters are driving Australian figures even during their lockdown: a hospital in Tasmania, an abattoir in Victoria and a nursing Home in New South Wales [9].

Transmission dynamics and containment strategies will look very different in selected settings, and this cannot be seen any more clearly than in facilities housing hundreds or thousands of individuals. Community engagement and other public health measures will need to be targeted and adaptable to the needs of these settings. Singapore’s case and contact finding measures paired with isolation and quarantine were extremely effective at stopping community spread. The same principles are now being adapted and tailored to fit the needs of the dormitory residents, their living circumstances and the scale of the issue.

## Conclusions

The blunt COVID-19 figures reported across the globe represent complex and disparate transmission dynamics in different settings, which require real-world epidemiological understanding and tailored outbreak response strategies. In Singapore, the numbers of COVID-19 cases being reported from the dormitories appear to be levelling off [2], and with mobilisation of medical care onsite, isolation facilities and massive screening exercises, a downward trend seems to be within sight.

Migrant workers around the world have been among the most vulnerable groups affected by the pandemic [10, 11]. The transparency offered by Singapore, and the publicly available data which allowed Tariq et al. to complete their analysis, may now provide a blueprint for effectively adapting COVID-19 containment strategies in order to protect some of our most vulnerable populations around the globe.

## Data Availability

Not applicable

## Abbreviations

COVID-19: Coronavirus disease 2019
Rt: Effective reproduction number
